# Sleep Disturbance in Older Patients in the Emergency Department: Prevalence, Predictors and Associated Outcomes

**DOI:** 10.3390/ijerph16193577

**Published:** 2019-09-25

**Authors:** Helen Mannion, D. William Molloy, Rónán O’Caoimh

**Affiliations:** 1 Clinical Sciences Institute, National University of Ireland, H91 TK33 Galway City, Ireland; h.mannion2@nuigalway.ie; 2 Centre for Gerontology and rehabilitation, University College Cork, St Finbarr’s Hospital, T12 XH60 Cork City, Ireland; w.molloy@ucc.ie; 3 Mercy University Hospital, T12 WE28 Cork City, Ireland

**Keywords:** sleep, emergency department, hospital, length of stay, frailty

## Abstract

Impaired sleep is common in hospital. Despite this, little is known about sleep disturbance among older adults attending Emergency Departments (ED), particularly overnight-boarders, those admitted but housed overnight while awaiting a bed. Consecutive, medically-stable patients aged ≥70, admitted through a university hospital ED were evaluated for overnight sleep quality (Richards Campbell Sleep Questionnaire/RCSQ) and baseline sleep (Pittsburgh Sleep Quality Index/PSQI). Additional variables included frailty, functional and cognitive status, trolley location, time in ED and night-time noise levels. Over four-weeks, 152 patients, mean age 80 (± 6.8) years were included; 61% were male. Most (68%) were ED boarders (*n* = 104) and 43% were frail. The majority (72%) reported impaired sleep quality at baseline (PSQI ≥ 5) and 13% (20/152) had clinical insomnia. The median time spent in ED for boarders was 23 h (Interquartile ± 13). After adjusting for confounders, median RCSQ scores were significantly poorer for ED boarders compared with non-boarders: 22 (± 45) versus 71 (± 34), respectively, (*p* = 0.003). There was no significant difference in one-year mortality (*p* = 0.08) length of stay (LOS) (*p* = 0.84), 30-day (*p* = 0.73) or 90-day (*p* = 0.64) readmission rates between boarders and non-boarders. Sleep disturbance is highly prevalent among older adults admitted through ED. ED boarders experienced significantly poorer sleep, without this impacting upon mortality, LOS or re-admission rates.

## 1. Introduction

While sleep disturbance is common in the general population, [1] it particularly affects older people [2]. Acute hospitals, like any similarly structured institution, are inherently ‘insomniogenic’ with high point prevalence rates of reduced (subjective) quality and quantity of sleep among inpatients, significantly worse than their usual sleep [3]. Markers of impaired sleep are independently associated with age-associated conditions including dementia [4] and frailty [5,6]. Exhaustion, a marker of frailty, results in an increased total cost of healthcare in older adults [7]. Sleep deprivation is also linked with cognitive dysfunction [8,9], delirium [10], metabolic disturbance [11] and immune deficiency [12]. Similarly, interventions targeting sleep may improve healthcare outcomes among older adults with [13] and without [14] cognitive impairment. Despite the associations between sleep dysfunction and adverse outcomes among older people, the impact of acute sleep disturbance on older hospitalised patients including those with differing levels of frailty is unclear. 

Most studies investigating the effects of sleep disruption in hospitals have focused on intensive care units (ICU), where evidence suggests that it is common, influenced by noise and may result in long-term sleep disorders after discharge [15]. Recently, a large prevalence study among inpatients in the Netherlands showed that sleep disruption is common on general hospital wards [3]. Sleep disturbance in the emergency department (ED) is also considered to be commonplace [16], particularly among those that stay overnight in the ED, while awaiting admission to an inpatient bed, often referred to as ‘ED boarders’ [17,18]. Despite this, the prevalence is not known and is difficult to determine given that healthcare systems strive that no patient should sleep overnight in the ED, though in many they do [16,18]. 

To date, little research into the prevalence of sleep disorders, disruption, associated factors or the effects of sleep disturbance in ED have been conducted including among vulnerable healthcare users. Understanding this complex picture and the differential effects of sleep disruption on the oldest and frailest cohort of ED users is important to plan and improve delivery of care. In particular, it is unknown whether sleep impacts on hospital length of stay (LOS), readmission rates, mortality or patient quality of life (QOL). Given these, the aims of this study were to assess the prevalence and factors associated with sleep disturbance among older patients admitted through an university hospital ED, to investigate if there is an impact of poor sleep on these patients, and to compare outcomes of ED boarders to those admitted directly to an inpatient bed on their first night in hospital.

## 2. Materials and Methods

### 2.1. Patients

Consecutive patients admitted to a single, large university hospital (University Hospital Galway in the West of Ireland) were included over a four-week period beginning in the summer of 2018. Included patients were aged ≥70 years, medically stable based on a Manchester Triage System (MTS) score > 1 [19], deemed suitable for assessment by medical or nursing staff and able to provide informed written consent. Those admitted directly to a critical care bed (acute stroke, ICU, coronary care or other high-dependency units) were excluded. The ED at University Hospital Galway had 64,096 attendances including 9407 aged ≥70 in 2016. Ethics approval was obtained from the local ethics committee of Galway University Hospitals (reference number C.A. 2015).

### 2.2. Data Collection

Data were collected for four weeks from Monday to Friday during core working hours (between 9.00 and 17.00) by a trained research assistant. Patients admitted during the preceding 24 h were evaluated the next morning. Those boarding in ED overnight and those admitted directly to a bed on a ward after attending ED including via the hospital’s acute medical assessment unit (AMAU) were included The initial two-weeks of data collection included only those admitted through the ED but as few medically stable patients were admitted directly from ED, those admitted via the AMAU having presented to ED triage were also included. Only patients spending at least six hours (between 10 pm and 8 am) in a bed during the first 24 h of their admission were regarded as having been admitted directly to a bed. Patients were evaluated using a study-specific questionnaire comprising various validated sleep measures and other scales assessing cognition, mood, functional level and frailty status. Medical records were reviewed to obtain demographics, past history and current prescription medications including hypnotics used to promote sleep. In addition, the time spent in ED, trolley location (main corridor, main cubicle area or other) and reasons for sleep disturbance were documented. Noise (sound pressure levels) in ED and AMAU were measured objectively with a smart phone application (Decibel X-Sound Meter dBA, Noise Detector, © SkyPaw Co. Ltd, Hanoi, Vietnam) based on the average of three recordings taken at 3 am at three locations in ED and one location in the AMAU short-stay ward on the three consecutive nights during the study. Between three and five days after admission those who remained inpatients were followed up and asked regarding change in sleep and QOL. Outcomes including total LOS (from the decision to admit in ED or AMAU), one-year mortality and 30 and 90-day readmission rates after discharge (excluding elective admissions), were obtained through ED case records and the Patient Administration System. 

### 2.3. Sleep Measures

The following measures of sleep were collected: The Richard Campbell Sleep Questionnaire, (RCSQ) [20], Pittsburgh Sleep Quality Index (PSQI) [21,22] and Insomnia Severity Index (ISI) [23]. The RCSQ is a validated, reliable [24] survey initially designed to measure subjective overnight sleep quality in an ICU setting [20]. This tool comprises five aspects: sleep depth, latency (time to achieve sleep) number of awakenings, efficiency (proportion spent awake), and sleep quality, scored on a 100 mm 10-point visual analogue scale (VAS). The mean of the five scales gives a total score for overnight sleep; higher scores suggest better sleep. For this study, a score of <70 was regarded as poor overnight sleep, in keeping with previous studies [25]. The PSQI is a 19-item questionnaire detailing different aspects of sleep, providing a reliable, valid, standardised measure of subjective sleep quality [21]. Widely used in different clinical and non-clinical settings [26], it discriminates poor sleep from good sleep over the preceding 30-days and is an easy-to-use tool for patients and clinicians alike. Higher scores indicate poorer estimated baseline sleep and a cut-off score of ≥5 from a total maximum of 19 points [21] was used to denote recent poor sleep quality in this study. The ISI is a brief, validated, seven-question self-reported scale used to evaluate patient perception of daytime and night-time symptoms of insomnia [23]. Scored out of 28 points, baseline insomnia was defined by an ISI score of ≥15 [23]. Finally, patients were asked to complete a five-point Likert scale rating their overnight sleep (global subjective assessment), “How would you rate your quality of sleep?” from one (very poor) to five (very good). This was used as the response variable against which the optimal cut-off score on the RCSQ was calculated, dichotomizing answers into poor (very poor and poor) and satisfactory (average, good, very good). 

### 2.4. Other Measures

In addition, a selection of rater and self-administered assessment scales were collected. Frailty was screened using the Programme on Research for Integrating Services for the Maintenance of Autonomy 7 item (PRISMA-7) questionnaire [27]. Individuals screen positive for frailty if they score ≥3 of the seven questions. The Clinical Frailty Scale (CFS) [28] is a subjective picture-based frailty scale, scored from one (fit) to nine (frail and terminally ill), which can be used to identify frailty, predict increased LOS and subsequent inpatient mortality [29]. QOL was measured using the Euroqol EQ-5D including a 100-point VAS self-rated from zero, an individual’s worst imaginable health state to 100, their best, at the present moment [30]. This was administered the morning after and at 3–5 days if patients remained as inpatients. Delirium was screened using the 4AT [31], a widely validated 4-question rapid test for delirium; scores of 1–3 suggest possible cognitive impairment, a score of ≥4 possible delirium [31]. The 20-point Barthel Index was used to score basic activities of daily living (ADL); the Barthel is scored from zero suggesting functional dependence to twenty indicating independence in personal ADL [32] including mobilising, transferring, continence, bathing, grooming, dressing, eating and managing stairs. Depression was evaluated with the five-question AB Clinical Depression Screen (ABCDS) [33], which incorporates items from the Geriatric Depression Scale. Co-morbidity was scored on the widely-validated Age-adjusted Charlson Co-morbidity Index (CCI) [34]. It measures burden of disease (17 co-morbidities weighted by severity) and one-year mortality with scores ≥5 indicating 85% one-year mortality and a high burden of co-morbidity [35]. 

### 2.5. Statistical Analysis and Sample Size Calculation

Data were analysed using SPSS V 24.0 (IBM, Chicago, IL, USA). The Kolmogorov-Smirnov test and Q-Q plots were used to test normality. Most data were non-normally distributed. Spearman’s correlation coefficient was used to assess inter-rater reliability (IRR) for a random sample of five patients comparing the trained rater and a consultant geriatrician with an interest in sleep and ageing. The Mann–Whitney U test or the Student t test compared independent samples and the Chi Squared test was used to compare distributions between variables. Binary logistic regression was used to explore the strength of the relationship between variables. The maximal area under the curve (AUC) of receiver operating characteristic curves was used to calculate the optimal cut-off for the RCSQ [36]. Approximately, one third of all adults report sleep disturbance [1] with 70% of older adults, aged ≥ 65 years, reporting at least one symptom of insomnia [37]. While the proportion of older adults with sleep disturbance (including symptoms of insomnia and poor overnight sleep quality) among those admitted to hospital and boarding overnight in ED is not known, we calculated this based on an assumed prevalence of 70%. Using the following formula, *n* = Z^2^P(1 − P)/d^2^ [38], where *p* = expected prevalence (70% as a proportion = 0.70), Z = level of confidence (1.96) and *d* = precision (between 5 and 10% = 0.05–0.1, given resource limitations we examined both), we estimated that the sample size required to power the study would be between 81 and 322 at a precision of 10% and 5%, respectively.

## 3. Results

### 3.1. Baseline Characteristics

**Figure 1:**
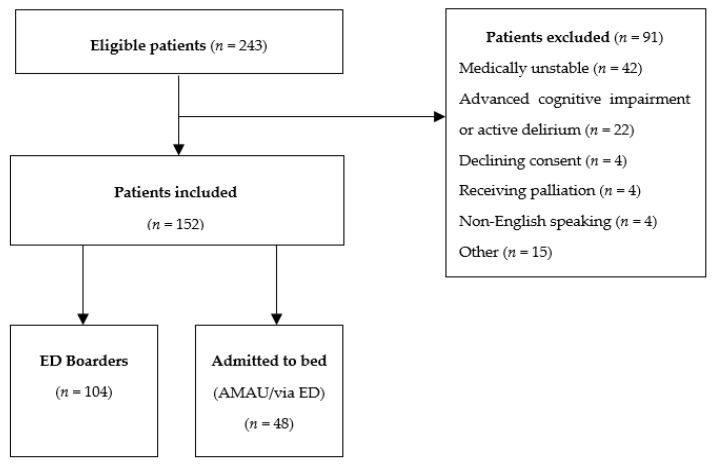
Flow diagram detailing patient selection including those boarding in the Emergency Department (ED) or admitted directly from ED or from the Acute Medical Assessment Unit (AMAU).

In all, 243 patients were approached of whom 152 were included. Patient selection is presented in Figure 1. Reasons for exclusion included being medically unsuitable as judged by medical or nursing staff on duty (*n* = 42), having advanced cognitive impairment or an acute confused state suggesting hyperactive delirium (*n* = 22), declining consent (*n* = 4), receiving palliation (*n* = 4), being non-English speaking (*n* = 4) and others (*n* = 15). There were no differences in age (*p* = 0.11) or MTS scores (*p* = 0.23) between those included and excluded, though most of those excluded (57%) were female and most (61%) included were male (n = 92) (*p* = 0.007). The mean age of those finally included was 80 years, standard deviation (SD) ± 6.8, a median of 80 years, interquartile range (IQR) ± 10. All patients were stable based on the MTS, median score was 3 (IQR ± 0). There was a high prevalence of frailty with 43% scored as frail using a CFS cut-off of ≥5 (mean CFS score 4.23 SD ± 1.6); a further 24% scored 4, indicating pre-frailty. Multi-morbidity was also common in this sample; the mean CCI score was 6 (IQR ± 2). Most patients were functionally independent in personal ADL at baseline (median Barthel Index score 19 IQR ± 5). Based on a 4AT cut-off of ≥4, 7% (*n* = 11) had possible delirium; 18% (*n* = 27) of patients scored between 1–3 points, suggesting cognitive impairment. While polypharmacy, defined as the current prescription of five prescription medications or more [39] was common (86%), only 14 (9%) were currently prescribed a hypnotic (4/14 were benzodiazepines), none of which were stopped on admission. Most patients reported having a high QOL based on a median self-reported Euroqol EQ-5D VAS score of 70 (IQR ± 22), indicating overall good health.

### 3.2. ED Boarders 

The majority of the sample (68%) were ED boarders (*n* = 104). The remainder (*n* = 48), apart from four patients admitted directly to beds from ED without ‘boarding’, were admitted from the AMAU. Approximately one-third of those staying overnight in ED boarded in the corridor of ED (32%); the remainder boarded on trolleys in cubicle areas at differing distances from the nurses’ station. The median time spent in ED for boarders was 23 h (IQR ± 13) and the median duration slept was just one hour (IQR ± 3, range 0–8). Baseline characteristics for the total sample and the comparison between ED boarders and those admitted directly to a hospital bed are presented in Table 1. 

### 3.3. Prevalence of Sleep Disturbance

**Figure 2:**
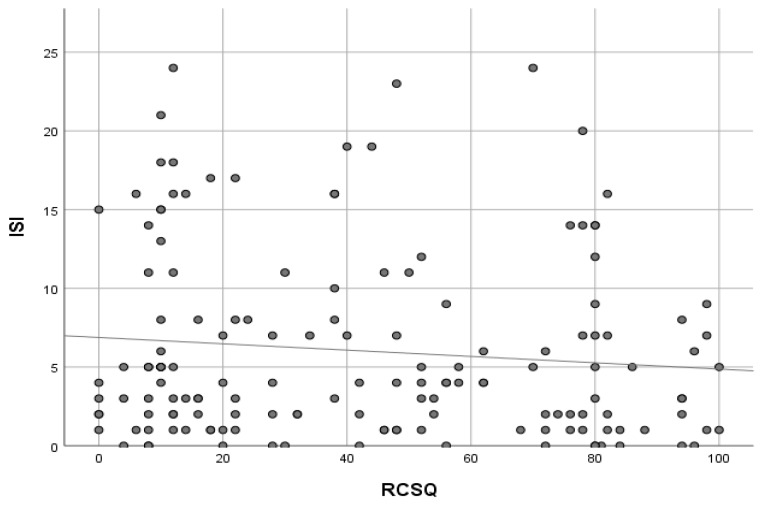
Scatterplot showing the correlation (*r*= −0.1) between baseline clinical insomnia judged by Insomnia Severity Index (ISI) scores and self-reported overnight sleep quality based on Richard Campbell Sleep Questionnaire (RCSQ) scores (*n* = 152).

**Figure 3:**
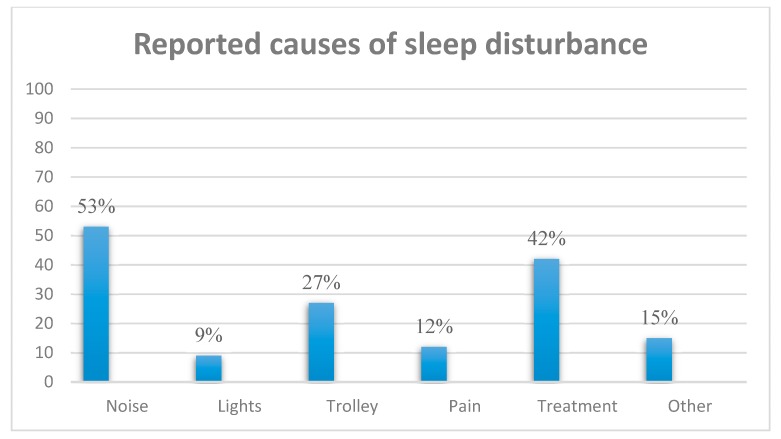
Self-reported causes for sleep disturbance for patients boarding overnight in the emergency department.

Baseline PSQI and ISI scores are presented in Table 1. IRR for the sleep instruments was excellent with strong correlation between the rater and expert for all three measures: PSQI (*r* = 0.83), ISI (*r* = 0.86) and RCSQ (*r* = 0.98). The majority of all patients, 72% (110/152), reported impaired sleep quality at baseline (PSQI ≥ 5) and 13% (20/152) had clinical insomnia as defined by the ISI (≥15). For overnight sleep in the hospital, the majority reported poor sleep on the first night of admission based on the RCSQ (<70 = poor sleep), 71% (108/152). Self-reported overnight sleep quality was significantly poorer for ED boarders compared with non-boarders: median RCSQ scores of 23 (IQR ± 45) versus 71 (IQR ± 34), respectively (*p* < 0.001). Based on the maximal AUC approach, the optimal cut-off on the RCSQ (using the global subjective measure of overnight sleep as a comparator), was <34, which gave a sensitivity of 87% and specificity of 80% for poor overnight sleep: AUC of 0.89, 95% CI: 0.82–0.96. While the ISI and PSQI correlated strongly (*r* = 0.79), a history of clinical insomnia at baseline did not appear to influence overnight sleep quality; ISI scores correlated weakly (*r* = −0.10) with RCSQ scores (see Figure 2). After adjusting for potential confounders (age, gender, use of hypnotic medications, MTS, ISI, PSQI, 4AT, Barthel Index, Charlson and CFS scores with one degree of freedom), ED boarders reported statistically significantly worse sleep (*p* = 0.003). Boarding in ED was associated with a greater than fourfold increased likelihood of reporting poor overnight sleep (odds ratio 4.36), see Table 2. This was confirmed using a simple 5-point Likert scale administered to those boarding in ED; 70% (73/104) scored their overnight sleep as poor or very poor. Based on a RCSQ cut-off to <34, ED boarding remained the only independent predictor of poor overnight sleep (p<0.001), OR 5.6 (95% CI 2.14−14.7). The most commonly reported causes of sleep disturbance were noise (53%) and interruptions for nursing or medical interventions (42%), (see Figure 3). Noise levels in ED and AMAU were high, mean score of 61.3 Decibels (dB) ranging between 63.1 and 68.5 dB in ED. Levels were significantly higher in ED compared with the AMAU (64.5 ± 1.8 dB vs. 51.7 ± 2.9 dB, respectively, *p* < 0.001). Neither the noise level or the location of boarding in ED (cubicle, corridor or other) were associated with overnight sleep quality (*p* = 0.31).

### 3.4. Sleep and Adverse Outcomes 

ED boarders reporting poor or very poor sleep on the global Likert scale decreased markedly and statistically significantly from 70% to 34% (*p* < 0.001) on follow-up. ED boarders reported lower self-reported QOL using the Euroqol EQ-5D VAS, albeit this was of borderline statistical significance (*p* = 0.06). Follow-up VAS results also showed no significant difference between boarders and non-boarders (*p* = 0.37), indicating that there was no effect on QOL. The median LOS of patients included in this study was six days (IQR ± 8). At one year 15 (10%) were dead. The median 30-day readmission rate was 44/152 (29%) and 90-day readmission rate 67/152 (44%). There were no statistically significant differences in median LOS, 6 (± 7) vs. 7 (± 8) days (*p* = 0.17), one-year mortality 7% vs. 17% (*p* = 0.08), 30-day readmission rates 31/104 (30%) vs. 13/48 (27%) (*p* = 0.73), or 30-day readmission rates 44/104 (42%) vs. 23/48 (48%) (*p* = 0.64) between boarders and non-boarders, respectively. Similarly, those with clinical insomnia (ISI ≥ 15 at baseline), poor recent sleep (PSQI baseline ≥ 5) and overnight sleep quality (RCSQ ≥ 70), irrespective of location, did not have statistically significant differences in these outcomes. 

## 4. Discussion

This study presents the prevalence of sleep disturbance (sleep quality and clinical insomnia) at baseline and after admission to hospital (overnight sleep quality) comparing rates between those boarding in ED overnight and those admitted directly to a bed on a ward after attending ED. The results show that there is a high prevalence of existing sleep problems among all older adults (aged ≥ 70 years) admitted to hospital with the majority (72%) reporting poor sleep and a relatively large proportion (17%) reporting clinical insomnia at baseline (prior to admission). This study found that most older adults admitted to a typical Irish university hospital ED spend a significant proportion of their initial 24 h in hospital in ED, sleeping for a time on a trolley; here the median time spent in ED boarding was 23 h with a median duration asleep of just one hour. These, ‘ED boarders’ reported having very little sleep and poor overnight quality and as would be expected, they had significantly lower quality of overnight sleep (RCSQ scores) compared to similar patients admitted directly to a bed (non-significant differences in age, sex, frailty, CCI and MTS scores). The odds of them reporting poorer sleep were 4.4 times higher than those admitted directly to a bed. In multivariate analysis pre-existing clinical insomnia did not influence self-reported overnight sleep quality and there was weak correlation between the ISI and RCSQ, indicating that ED’s are truly ‘insomniogenic’. Boarders reported that ED is noisy at night with interventions (e.g., blood pressure readings) as well as physical discomfort (pain and bright lights) disturbing their sleep. This was corroborated objectively with noise levels, which were significantly higher in ED compared with the AMAU and were similar to other EDs in countries including Brazil [40] and the USA [41]. By comparison a noise level of 65 dB is equivalent to piano practice and 70 dB to standing under a hair dryer. On follow-up, once ED boarders had received a bed, subjective global sleep (measured on a Likert scale) improved significantly. It is unclear if this relates to the change of environment (e.g., reductions in noise) or improvements in overnight sleep quality because of changes in health status (recovery from illness) over time. This did not change self-reported QOL. Despite poor self-reported sleep, those boarding in ED did not experience a greater incidence of adverse outcomes and had similar LOS and readmission rates to non-boarders. Similarly, measures of sleep disturbance (ISI, PSQI and RCSQ) were not associated with these outcomes. 

These results compare favorably with another study in a large ED in Ireland (Dublin), which showed that older adults had the longest waiting times and that boarding in ED did not impact on adverse outcomes such as inpatient mortality [18]. That study did not however, measure or include sleep in its modelling. Our study also compares with the only previous and recently published paper examining causes of self-reported poor sleep in a Canadian ED (Montreal), finding that stress, noise, pain, and trolley comfort were associated with lower sleep quality, which was 20% lower than in the week before admission [16]. That study did not use a validated measure of sleep quality, included adults aged ≥ 18 years and used convenience sampling [16], limiting comparability to this study. To date, few studies have examined the prevalence of sleep disturbance in acute hospitals and none to our knowledge have examined the prevalence in sleep disturbance in the ED. A recent Dutch study showed that inpatients on hospital wards across the Netherlands (57% aged ≥ 65) reported significantly reduced sleep after admission with pain and noise from other patients and medical devices identified as associated factors [3]. Patients were only included if they had spent at least one night in a hospital bed and no impact of time spent in ED was conducted [3]. Another study of adult inpatients in a general hospital in China (Tiajin) using the PSQI found that 45.6% reported poor sleep quality during hospitalisation [42]. Similarly, a study conducted in the United States found that 36% of inpatients in a general hospital ward developed new insomnia based on the ISI; staff interruption, their physical condition (e.g., pain) and noise identified as the most common reasons for sleep disruption [43]. 

### Strengths and Limitations

This study is, to our knowledge, the first to examine the prevalence of sleep disturbance among older adults admitted to ED. Consecutive sampling was used to obtain a representative sample of older adults and to measure prevalence based on a comprehensive battery of recognised and well-validated sleep measures. This study has a number of limitations. Due to time constraints, data collection was conducted during core working hours only, potentially resulting in sampling bias. Time and resource constraints also affected the sample size, likely underpowering the study: using the more appropriate precision for prevalence studies, where the expected prevalence is between 10–90%, i.e., 5%, then the sample size obtained was insufficient [38]. This said, the exact population prevalence of sleep disturbance including poor overnight sleep among older adults admitted to hospital, particularly among those boarding in ED, is not known and may be higher than 70%. Hence, this study should be considered as a pilot study and the results could be used to inform a future sample size calculation. In addition, those deemed critically unwell based on the MTS or requiring critical care were excluded adding to the risk of sampling bias. There was variation in the timing of follow-up, though all patients were reassessed within the target of five days. The follow-up assessment was limited in scope with a focus on subjective measures of QOL and overall (global) sleep quality. Hence, the RCSQ was not re-administered, which limits the ability to show that overnight sleep quality improved when patients slept outside of ED in a hospital bed on a ward. Changes to the use of hypnotics during admission were not recorded and would be important to include in future studies. Other research shows that hospitalisation increases the prescription of hypnotics, particularly within the first 24 h of admission and this leads to an elevated risk of drug-drug interactions and other drug-related problems [44]. Likewise, no follow-up of the long-term impact on sleep was conducted. This said, the primary focus of the study was to assess the prevalence of sleep disturbance among older people in ED. Another limitation concerns the selection of instruments used to measure sleep; no objective measures such as actigraphy were used. The PSQI, while valid and reliable among older adults [45,46], is affected by the same problems as other self-report inventories in that scores can be easily exaggerated or minimized by the person completing them [47]. Similarly, as with all questionnaires, the way the instrument is administered can have an effect on the final score, potentially leading to reporting bias. This said, IRR testing showed that scoring was reliable. Furthermore, the PSQI, ISI and RCSQ are relatively new measures and as a result have not been investigated to determine the entirety of their psychometric properties. Study is required to assess these with older adults in an ED setting. Examination of the long-term effects of boarding in ED on sleep and other outcomes also merits further study, particularly as sleep deprivation and sleep-wake cycle disturbances while an inpatient likely contribute to an increased risk of adverse events, the so called ‘post-hospital syndrome’ [48].

## 5. Conclusions

Given the growing focus on patient satisfaction and the rise of hospital league tables in many countries, understanding the prevalence, predictors and impact of sleep disturbance on patients while in hospital will become increasingly important. Indeed, better categorisation of adults attending EDs is required to improve decision-making for these patients [49]. The initial 24 h after admission are an important time in the care of patients in acute care settings and a high proportion of these spend it in ED. While no healthcare systems strive to house people overnight in EDs, this remains a clinical reality for many hospitals struggling to address challenges associated with overcrowded EDs and is recognised as an international phenomenon [18]. Our study, the first to use established sleep questionnaires in ED to measure the prevalence of sleep disturbance, shows that impaired sleep is common among older people. While ED is a difficult environment to intervene and improve sleep in, simple approaches to reducing noise (including the use of ear plugs) and alterations in light have proven effective [50,51]. Adequately powered studies and in other sites are now required to investigate the true prevalence of sleep disturbance amongst older adults admitted via ED, to assess if this impacts on clinical outcomes and healthcare costs and to evaluate if interventions can mitigate these.

## Figures and Tables

**Table 1:** Baseline characteristics of patients comparing those admitted directly to a hospital bed including via the acute medical assessment unit (AMAU) to those boarding in the emergency department (ED).

Variable	Total (*n* = 152)	‘Direct’ to Bed (Including AMAU) (*n* = 48)	ED Boarder (*n* = 104)	*p* = X
**Age (years)** **Median ± IQR**	80 (84 − 74= ± 10)	81.5 (86 − 76 = ± 10)	79 (83 − 74 = ± 9)	*p* = 0.11
**Gender (% M)**	61%	73%	57%	*p* = 0.49
**Polypharmacy** **(% ≥ Five medications)**	86%	87.5%	85%	*p* = 0.64
**Manchester Triage Score** **Median ± IQR**	3 (3 − 3 = ± 0)	3 (3 − 3 = ± 0)	3 (3 − 3 = ± 0)	*p* = 0.82
**RCSQ** **Median ± IQR** **(Poor sleep overnight < 70)**	40 (75 − 12 = ± 63)	71 (80 − 46 = ± 34)	23 (55 − 10 = ± 45)	*p* ≤ 0.001
**PSQI score** **Median ± IQR** **(Poor sleep quality at baseline ≥5 )**	7 (11 − 4 = ± 7)	7.5 (11 − 4 = ± 9)	7 (11 − 4 = ± 7)	*p* = 0.73
**ISI score** **Median ± IQR** **(Insomnia at baseline ≥15)**	4 (8 − 2 = ± 6)	4 (9 − 2 = ± 7)s	4 (8 − 2=±6)	*p* = 0.79
**Euroqol EQ-5D VAS (QoL)** **Median ± IQR**	70 (80 − 58 = ± 22)	72.5 (80 − 60 = ± 20)	70 (80 − 50 = ± 30)	*p* = 0.06
**Barthel Index** **Median ± IQR**	19 (20 − 15 = ± 5)	18 (20 − 1 5= ± 5)	19 (20 − 15 = ± 5)	*p* = 0.61
**4AT** **Median ± IQR**	0 (1 − 0 = ± 1)	0 (0 − 0 = ± 0)	0 (0 − 0 = ± 0)	*p* = 0.84
**ABC Depression Score** **Median ± IQR**	0 (0 − 0 = ± 0)	0 (0 − 0 = ± 0)	0 (0 − 0 = ± 0)	*p* = 0.89
**PRISMA-7** **Median ± IQR**	4 (6 − 2 = ± 4)	4 (6 − 3 = ± 3)	4 (5 − 2 = ± 3)	*p* = 0.17
**Charlson Co-morbidity Index** **Median ± IQR**	6 (7 − 5 = ± 2)	6 (8 − 5 = ± 3)	6 (7 − 5 = ± 2)	*p* = 0.29
**Clinical Frailty Scale** **Median ± IQR** **(% Frail: CFS ≥ 5 - at assessment)**	4 (5 − 3 = ± 2) (43%)	5 (5 − 3 = ± 2) (49%)	4 (5 − 3 = ± 2) (42%)	*p* = 0.29

IQR = Interquartile range; ISI = Insomnia Severity Index; PRISMA-7 = Programme on Research for Integrating Services for the Maintenance of Autonomy 7 item; PSQI = Pittsburgh Sleep Quality Index; RCSQ = Richard Campbell Sleep Questionnaire; VAS = Visual Analogue Scale; 4AT = 4 A’s Test.

**Table 2:** Binary logistic regression model showing the association, odd ratio (OR), between variables including boarding in the emergency department (ED) and poor overnight sleep based on a Richards Campbell Sleep Questionnaire cut-off score of <70.

Variable	OR	95% Confidence Interval	*p* = x
Age	1.05	0.98–1.13	0.14
Barthel Index	0.99	0.85–1.15	0.88
Boarder in ED	4.36	1.67–11.4	0.003
Clinical Frailty Scale	0.69	0.40-1.19	0.18
Charlson Co-Morbidity Index	0.90	0.63–1.30	0.59
Use of hypnotic medications	0.40	0.10–1.67	0.21
Gender (male)	0.90	0.36–2.26	0.83
Insomnia Severity Index	1.00	0.92–1.09	0.96
Manchester Triage System score	1.79	0.53–6.05	0.35
Pittsburgh Sleep Quality Index	2.41	0.78–7.87	0.15
4 AT score	0.93	0.69–1.26	0.64
